# Developing a syndromic surveillance tool to support the epidemiological investigation into paediatric acute hepatitis of unknown aetiology in England

**DOI:** 10.1186/s12889-025-23222-0

**Published:** 2025-05-31

**Authors:** Alex J. Elliot, Helen E. Hughes, Christopher Bennett, Thomas C. Hughes, Kirsty Challen, Conall H. Watson, Sema Mandal, Gillian E. Smith, Daniel Todkill

**Affiliations:** 1 Real-time Syndromic Surveillance Team, Field Services Division, UK Health Security Agency, Birmingham, UK; 2 Oxford University Hospitals NHS Foundation Trust, Oxford, UK; 3 Lancashire Teaching Hospitals NHS Foundation Trust, Preston, UK; 4 Immunisation and Vaccine Preventable Diseases Division, UK Health Security Agency, London, UK; 5 Blood Safety, Hepatitis, STI and HIV Division, UK Health Security Agency, London, UK

**Keywords:** non-HepA–E hepatitis, Hepatitis of unknown origin, Paediatric acute hepatitis of unknown aetiology, Acute hepatitis, Paediatric hepatitis, Syndromic surveillance, Emergency department

## Abstract

**Background:**

During 2022, a new public health threat emerged when cases of paediatric acute hepatitis of unknown aetiology (HUA) were identified in children aged under 16 years old in the United Kingdom (UK). At the time, the epidemiology and extent of cases was based upon limited and non-standardised reporting from hospitals and liver units. We aimed to adapt existing real-time syndromic surveillance systems to support the epidemiological investigation of cases of HUA presenting to emergency departments (EDs) in England.

**Methods:**

Syndromic surveillance is generally based on the collection of patient symptoms or chief complaints, which are collected using automated routines in near real-time. Here, we used an existing ED syndromic surveillance system monitoring daily patient attendances across a network of approximately 150 EDs in England. Clinical diagnosis codes related to the potential symptoms associated with the HUA incident were selected and attendance data monitored retrospectively and prospectively during the incident.

**Results:**

From 2 April 2018 to 31 December 2021, there were small sporadic numbers of daily ED attendances for ‘liver conditions’ in children with no observed secular trends or seasonality across the 1 to 4 and 5 to 14 years age groups. The period 2 April to 29 July was compared across each year included in the analysis. Mean daily HUA attendances during 2018 to 2021 was 0.05 and 0.22 for 1 to 4 and 5 to 14 years respectively, however in 2022 there were 0.26 and 0.42 mean daily attendances. This represented an increase of 377% and 94% in the 1 to 4 and 5 to 14 years age groups, respectively. From June 2022, daily syndromic ‘liver condition’ attendances appeared to decrease and the rate of increase in cumulative attendances slowed.

**Conclusions:**

We demonstrate how syndromic surveillance provided support to the HUA outbreak using an existing syndromic surveillance framework to develop new indicators based on the newly emerging clinical symptoms. The outputs from the syndromic tool matched clinical and epidemiological findings with respect to trends in other HUA-related data, including clinical and laboratory reports, over time. This work demonstrates the potential for syndromic surveillance supporting the epidemiological surveillance of hepatitis and providing a valuable tool for the real-time management of future unknown health threats.

## Introduction

As of 4 July 2022, 274 confirmed cases of paediatric acute hepatitis of unknown aetiology (HUA) had been identified in children aged under 16 years old in the United Kingdom (UK), of which 195 were resident in England [1]. In England, cases of HUA were first identified by the UK Health Security Agency (UKHSA) in March to April 2022, and on clinical lookback HUA cases were subsequently identified back to January 2022 [2–4]. Globally, the number of HUA cases increased dramatically to over 1000 cases at the start of July, reported across 35 countries [5, 6]. In the UK, overall 97% of cases were admitted to hospital and 5% required liver transplantation, however there were no UK deaths reported [1, 4]. Although this was perceived to be an outbreak that originated in 2022, there was evidence of HUA cases identified in the US in 2021 [7].

Surveillance is an essential tool for monitoring newly emerging threats, helping with initial identification of the threat, then providing timely situation awareness to inform on trends and burden to allow for effective public health action (including interventions) to be implemented. Adenovirus had been implicated in the aetiology of HUA cases [7–9], however during the outbreak there was no specific laboratory (or other) test to provide a ‘confirmed diagnosis’ of HUA and therefore other sources of data had to be considered for surveillance in this evolving hepatitis outbreak. ‘Syndromic surveillance’, utilising presenting symptoms and provisional clinical diagnoses, can potentially support the public health response to this incident. Here we demonstrate how we rapidly developed a real-time syndromic surveillance tool from within a pre-existing surveillance system, to support the national HUA incident response in England.

## Methods

“Syndromic surveillance is the real- (or near real-time) collection, analysis, interpretation, and dissemination of health-related data to enable the early identification of the impact (or absence of impact) of potential public health threats that require effective public health action” [10]. UKHSA routinely coordinates a suite of national syndromic surveillance systems including: emergency department (ED) attendances; general practitioner (GP) in-hours and out-of-hours consultations; ambulance dispatch calls; and NHS (National Health Service) telehealth calls and online assessments [11]. Each syndromic system is built upon the daily receipt of anonymised data which are monitored and interrogated on a daily basis, using epidemiological and statistical methods [12, 13].

We undertook a short ‘options appraisal’ of the UKHSA syndromic surveillance systems in collaboration with members of the UKHSA HUA Incident Management Team to assess which syndromic systems and indicators would be of most value for supporting the surveillance of HUA activity. Factors considered in this options appraisal included the likelihood of HUA cases presenting to a healthcare service encompassed within the syndromic systems and the presence of appropriate and available clinical coding in the syndromic system. EDs were considered to be one of the most likely healthcare services to receive suspected HUA cases and therefore the UKHSA Emergency Department Syndromic Surveillance System (EDSSS) was selected for the development of HUA surveillance capability [14].

The EDSSS is a real-time surveillance system monitoring attendances across EDs in England. The daily reporting of attendances is typically from 150 Type 1 EDs. Each day, anonymised information on attendances recorded in the previous 24 h is received by UKHSA, including (anonymised) demographics on the patient, information on the initial triage (severity) of the attendance on presentation at the ED, clinical information on the diagnosis and the ultimate disposal (discharge) of the attendance (e.g. home, admitted, deceased). Surveillance data from EDSSS are included in daily routine epidemiological analysis and interpretation of trends across multiple (all hazard) syndromic indicators (e.g. acute respiratory infection, myocardial infarction, heat/sun stroke). The resulting EDSSS outputs are used to inform UKHSA and the NHS of emerging threats, early warning of seasonal outbreaks and reassurance of lack of impact during mass gatherings.

In England, EDs use the Emergency Care Dataset (ECDS) for the collection and storage of patient care data as well as the transmission of required fields to a central point (NHS England). The ECDS uses a specifically designed constrained SNOMED coding dataset to support diagnoses of presenting patients, which subsequently underpins the EDSSS [15]. Diagnosis codes within ECDS were appraised for the potential specificity to presenting HUA cases, with suitable clinical diagnosis codes grouped into a ‘liver conditions’ syndromic indicator (Table 1). The new syndromic ‘liver conditions’ diagnosis indicator was applied to the live EDSSS database on 6 April 2022. At that time, liver conditions attendances were monitored prospectively, and for incident reporting and this report, attendances were retrospectively analysed. Attendance data during 2022 were compared to historical data over the years 2018 to 2021 from a subset of EDSSS EDs that had consistently reported throughout the whole study period of 2018 to 2022 (n = 83). This consistent cohort of EDs prevented variation in attendances numbers across the study period resulting from new EDs reporting, or existing EDs leaving the surveillance system across the study period. Two of the study years (2018 and 2022) were incomplete and therefore a shorter period of 2 April to 29 July (119 days) was selected across each study year to enable a direct comparison of attendances across each year.

**Table 1:** Emergency care dataset SNOMED code grouping used in the syndromic ‘liver conditions’ indicator and frequency (percent) of use of code in the indicator

Snomed code	Descriptor	Percent
128241005	Inflammatory disease of liver (disorder)	46%
59927004	Hepatic failure (disorder)	32%
39400004	Injury of liver (disorder)	16%
37871000	Acute infectious hepatitis (disorder)	3%
40468003	Viral hepatitis, type A (disorder)	1%
66071002	Viral hepatitis type B (disorder)	1%
773113008	Acute hepatitis caused by infection (disorder)	< 1%

## Results

**Fig. 1:**
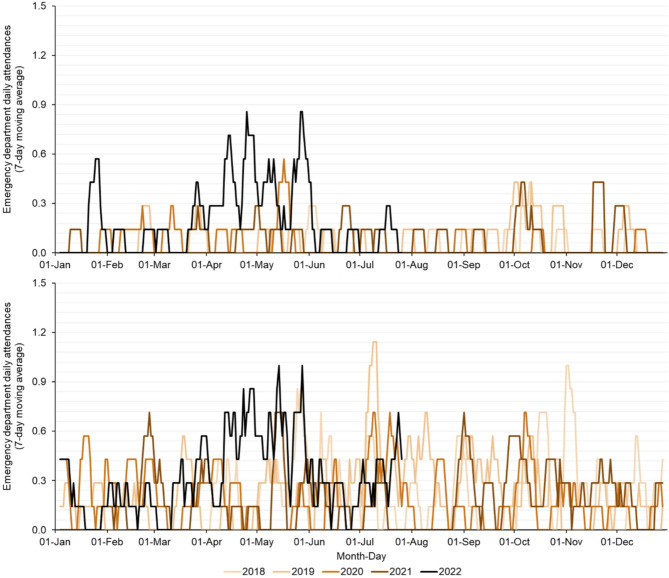
Daily emergency department attendances (seven day moving average) with a ‘liver condition’ primary diagnosis in children aged (**A**) 1 to 4 and (**B**) 5 to 14 years, 2 April 2018 29 July 2022 Note: contains public sector information licensed under the Open Government Licence v3.0

**Fig. 2:**
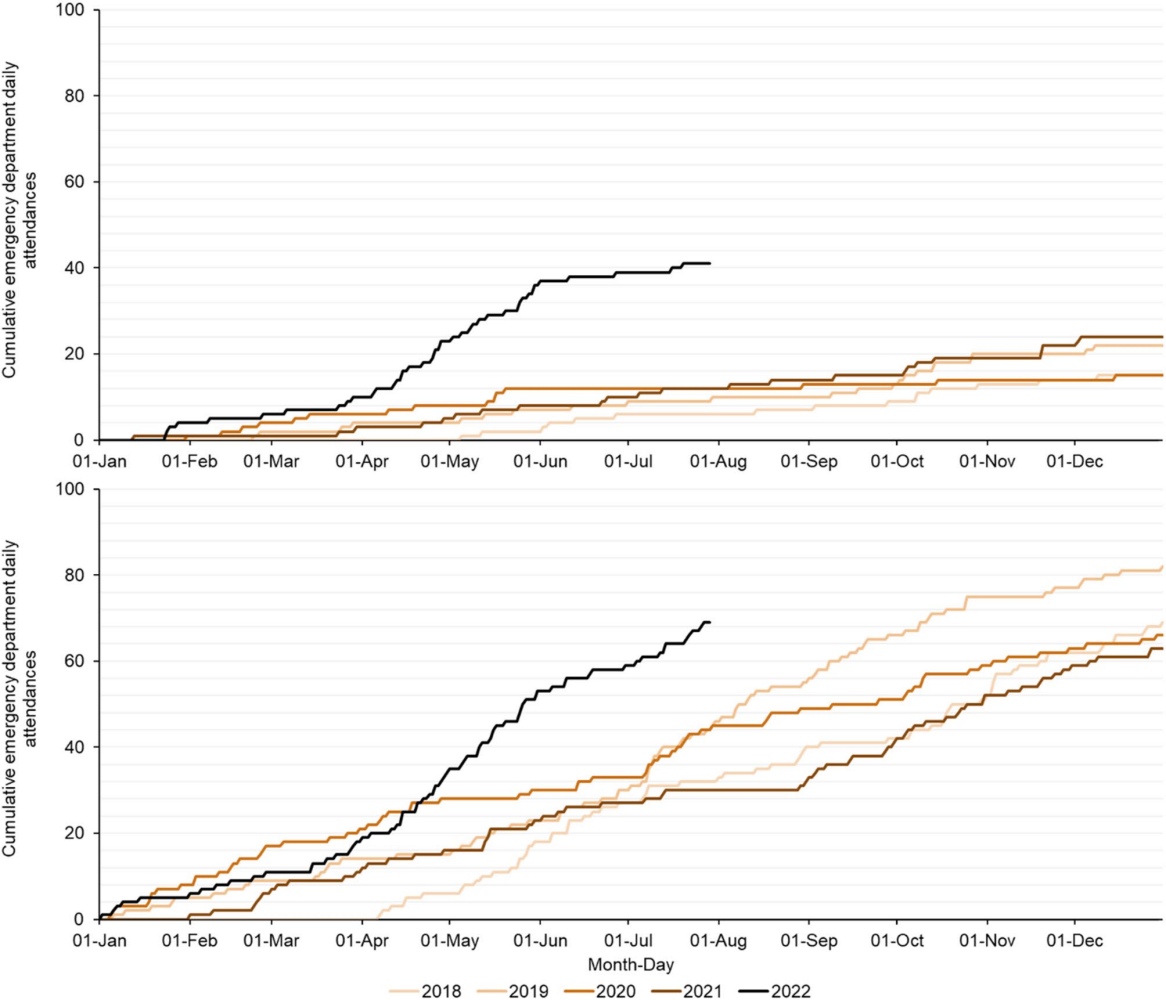
Cumulative daily emergency department attendances with a ‘liver condition’ primary diagnosis in children aged (**A**) 1 to 4 and (**B**) 5 to 14 years, 2 April 2018 to 29 July 2022 Note: contains public sector information licensed under the Open Government Licence v3.0

From 2 April 2018 to 31 December 2021, daily numbers of EDSSS attendances for ‘liver conditions’ were very small and sporadic, with very few attendances observed in the under 1 year age group (fewer than one attendance per month). There was no observed secular trend or seasonality across either the 1 to 4 or 5 to 14 years age groups (Fig. 1). Cumulative attendances over the calendar years 2019 to 2021 ranged between 15 and 24 annual ED attendances in children aged 1 to 4 and between 63 and 82 annual attendances in the 5 to 14 years age group (Fig. 2).

From 1 January to 29 July 2022 there were a total of 41 and 69 daily attendances reported in children aged 1 to 4 and 5 to 14 years, respectively. A period from 2 April to 29 July was compared across each year included in the analysis. During 2 April to 29 July, the mean daily HUA ED attendances over the years 2018 to 2021 was 0.05 and 0.22 for 1 to 4 and 5 to 14 years respectively. However, during the same period in 2022, 0.26 and 0.42 mean daily attendances were reported in the 1 to 4 and 5 to 14 years age groups, respectively. This represented a percentage increase of 377% and 94% in the 1 to 4 and 5 to 14 years age groups, respectively (Table 2). From June 2022, daily syndromic ‘liver condition’ attendances appeared to decrease in frequency, and the rate of increase in cumulative attendances slowed (Figs. 1 and 2).

**Table 2:** Emergency department liver condition attendances from 2018 to 2022 in children aged 1 to 4 and 5 to 14 years

**1 to 4 years**						
	2018	2019	2020	2021	2018 to 2021	2022
Liver condition attendances^a^	6	5	6	9	26	31
Mean liver condition attendances per day	0.05	0.04	0.05	0.08	0.05	0.26
% difference to 2022	417	520	417	244	377	N/A
Extrapolated annual liver condition attendances^b^	18	15	18	28	20	95
**5 to 14 years**						
	2018	2019	2020	2021	2018 to 2021	2022
Liver condition attendances^a^	32	30	23	18	103	50
Mean liver condition attendances per day	0.27	0.25	0.19	0.15	0.22	0.42
% difference to 2022	56	67	117	178	94	N/A
Extrapolated annual liver condition attendances^b^	98	92	71	55	79	153

^a^ liver condition emergency department attendances reported in a restricted period common across each study year (2 April to 29 July, 119 days)

^b^ liver condition attendances in the restricted study period (119 days) extrapolated to the full calendar year (365 days)

## Discussion

Here we present the use of syndromic surveillance to provide timely support for the epidemiological investigation into paediatric acute HUA in England, a novel emerging public health incident. Existing UKHSA national syndromic surveillance systems are established primarily to provide early warning of emerging threats, reassurance of a lack of threat and situational awareness during an emergency [10]. We used existing clinical coding available within the surveillance system to rapidly develop a new diagnostic syndromic indicator for ‘liver conditions’. These codes were identified as those most likely to be linked to potential HUA ED attendances based upon the emergency care expertise of the EDSSS working group. A new syndromic indicator based on these codes was implemented and used to support incident management [2].

EDSSS corroborated early reports of increases in HUA with an observed increase signal in syndromic liver condition attendances during early 2022 [2]. The syndromic indicator illustrated a large increase in attendances in children aged 1 to 4 and 5 to 14 years from March 2022, although the greatest burden in ED attendances was in the former age group. The syndromic data were used to supplement the incident management of HUA with the syndromic outputs included in technical briefings and used by incident management teams to better understand the epidemiology of the HUA outbreak. During June 2022, syndromic surveillance data informed on the potential slowing down of the outbreak in England thereby providing reassurance that the HUA had not escalated further. Overall, retrospective comparison of the HUA ED attendances with other sources of clinical and laboratory reported during the incident illustrated a good match in trends over time, supporting the prospective use of real-time syndromic surveillance to support this (and future) incident(s) [2].

This report further demonstrates the utility of syndromic surveillance and provides a coding toolkit for other users of syndromic surveillance to apply to local syndromic systems in the response to this global incident. Traditionally syndromic surveillance is used to monitor existing seasonal pathogens e.g. influenza, norovirus, however, the systems are flexible (encompassing presenting symptoms across all body systems e.g. gastrointestinal to neurological) and able to respond to new and emerging public health threats [10]. An advantage of utilising existing surveillance systems is the ability to compare contemporaneous data to historically expected baselines. The EDSSS has been operational for over 10 years, initially as a small sentinel system (2010 to 2018) and subsequently a large national system from 2018 [14, 16]. In this HUA incident, four years of historical data were selected from a subset of EDs who had consistently reported in the EDSSS, providing valuable context to understanding the impact of the emerging threat. Furthermore, EDSSS captures routinely available clinical data and therefore places no additional burden on frontline clinicians and remains unaffected by any changes in working pressures. EDSSS (and other syndromic surveillance systems in England) have to date not been used to support the epidemiological investigation and surveillance of acute viral hepatitis. However, the use of a bespoke ‘liver conditions’ indicator based on exiting clinical codes does encourage further exploration of whether syndromic surveillance can support or enhance existing public health surveillance of hepatitis to further understand its epidemiology.

EDSSS uses an internationally recognised clinical coding system (SNOMED) for the identification of diagnoses within the ED data received. The results from this work are therefore applicable in other countries using ED syndromic surveillance systems [17, 18]. In countries where HUA outbreaks have been identified, and incident management response established, syndromic surveillance can supplement the response. In those countries without cases, this syndromic approach may provide some reassurance of an absence of escalation of HUA.

However, it is important to note limitations with this method. It is likely that EDSSS did not capture the full burden of HUA ED attendances when compared to the incident management response case line list and therefore it cannot be used as an accurate record of the number of HUA cases. In addition, the comparison with previous years demonstrated that there was a varying number of ‘background’ (pre-incident) cases annually. Therefore, an important caveat when using syndromic surveillance is that it is an adjunct to, rather than replacing the need for, front-line ascertainment of cases. This study also used ED attendances collected over the COVID-19 pandemic. Previous work utilising the EDSSS showed the indirect impact that COVID-19 had on ED attendances [19]. Following the introduction of the first non-pharmaceutical interventions (social distancing and shielding) in England during March 2020, total ED attendances decreased by 47%, however there was a differential impact on respiratory and non-respiratory attendances, for example attendances for acute respiratory infections and gastroenteritis decreased by 4% and 67% respectively [19]. However, we feel that the impact of the pandemic on our study was minimal because of the more severe symptom presentation associated with ‘liver conditions’ in children. Parents or carers of children with these symptoms would not avoid presenting at an ED because of concerns about risks associated with visiting an ED during the pandemic.

The syndromic ‘liver conditions’ grouping is based on the recording preliminary clinical diagnostic codes used during the initial clinical assessment in the ED; further pathology results and examinations made outside the ED that identify HUA cases may result in under ascertainment of numbers. Furthermore, we adopted a ‘catch all’ syndromic coding approach by including clinical codes for ‘viral hepatitis, type A/B’. While we did not anticipate that a diagnosis of type A/B hepatitis would be commonly made in the ED setting, we wanted to ensure that any miscoding of hepatitis-like symptoms was captured in this indicator. However, it is possible that patients grouped into the liver conditions indicator may have been diagnosed subsequently (outside of the ED setting) as confirmed hepatitis cases. A further limitation of the syndromic approach is the potential impact of medical press and mainstream media coverage of the incident: increased awareness of the potential for cases of HUA may have influenced coding behaviour of clinicians who might be more disposed to using one of the ‘liver conditions’ clinical diagnosis codes when faced with a patient presenting with relevant symptoms. Furthermore, the public can also be more likely to present to an ED with associated symptoms because of heightened concern resulting from media coverage.

The ability to rapidly adapt existing data feeds to support the national public health response to an emerging threat is an important advantage of syndromic surveillance. One limitation of this approach is that for unforeseen clinical presentations, as seen in HUA, the bespoke groupings of codes are not prepared or routinely monitored and must therefore be developed post-identification of the threat. On retrospective analysis, increases in ED liver condition attendances were observed from early January 2022, however this cluster of ED codes had not been curated or monitored at the time of the emergence of HUA and therefore early warning of the incident through syndromic surveillance was not possible. We are now exploring the use of novel machine learning algorithms to understand how clusters of codes can be identified routinely, enabling syndromic surveillance systems to be used ‘agnostically’ and across all major body systems to create generic indicators for each e.g. liver, neurological, skin. This novel approach might signal an unusual threat before it is identified through traditional clinical, epidemiological or diagnostic pathways.

## Data Availability

The anonymised data included in this study are not publicly available. Applications for requests to access relevant data should be submitted to the UKHSA Office for Data Release. Available at: https://www.gov.uk/government/publications/accessing-ukhsa-protected-data (accessed 30 October 2023).
